# The Glycogen-Binding Domain on the AMPK β Subunit Allows the Kinase to Act as a Glycogen Sensor

**DOI:** 10.1016/j.cmet.2008.11.008

**Published:** 2009-01-07

**Authors:** Andrew McBride, Stephanos Ghilagaber, Andrei Nikolaev, D. Grahame Hardie

**Affiliations:** 1 Division of Molecular Physiology, College of Life Sciences, University of Dundee, Dundee DD1 5EH, Scotland, UK; 2 Division of Biological Chemistry and Drug Discovery, College of Life Sciences, University of Dundee, Dundee DD1 5EH, Scotland, UK

**Keywords:** HUMDISEASE, PROTEINS

## Abstract

AMPK β subunits contain a conserved domain that causes association with glycogen. Although glycogen availability is known to affect AMPK regulation in vivo, the molecular mechanism for this has not been clear. We now show that AMPK is inhibited by glycogen, particularly preparations with high branching content. We synthesized a series of branched oligosaccharides and show that those with a single α1→6 branch are allosteric inhibitors that also inhibit phosphorylation by upstream kinases. Removal of the outer chains of glycogen using phosphorylase, thus exposing the outer branches, renders inhibition of AMPK more potent. Inhibition by all carbohydrates tested was dependent on the glycogen-binding domain being abolished by mutation of residues required for carbohydrate binding. Our results suggest the hypothesis that AMPK, as well as monitoring immediate energy availability by sensing AMP/ATP, may also be able to sense the status of cellular energy reserves in the form of glycogen.

## Introduction

The AMPK-activated protein kinase (AMPK) is a regulator of energy balance at both the single-cell and whole-body levels (Kahn et al., 2005) and a target for drugs aimed at treatment of type 2 diabetes and the metabolic syndrome (Hardie, 2007a). AMPK is activated by metabolic stresses that inhibit ATP synthesis or accelerate ATP consumption and is modulated by cytokines regulating whole-body energy balance (Kahn et al., 2005). Once activated, the system switches on catabolic pathways that generate ATP while switching off ATP-consuming processes (Hardie, 2007b).

The native form of AMPK is a heterotrimeric complex comprised of a catalytic α subunit and accessory β and γ subunits. The γ subunits contain tandem domains that bind the regulatory nucleotides AMP and ATP in a mutually exclusive manner (Scott et al., 2004; Townley and Shapiro, 2007; Xiao et al., 2007). By inhibiting dephosphorylation, binding of AMP to the γ subunit promotes net phosphorylation of the kinase domain on the α subunit at a critical phosphorylation site in the activation loop (Thr-172), causing > 100-fold activation (Hawley et al., 1996; Suter et al., 2006). The phosphorylated kinase is also allosterically activated by AMP, with both effects of AMP being antagonized by high concentrations of ATP. Thr-172 is phosphorylated in most cells by the tumor suppressor kinase LKB1 (Hawley et al., 2003; Woods et al., 2003), but the effect of AMP on phosphorylation is due to its ability to bind to AMPK and render it a poorer substrate for protein phosphatases (Davies et al., 1995; Suter et al., 2006; Sanders et al., 2007). In some cell types (Hawley et al., 2005), Thr-172 can also be phosphorylated in a Ca^2+^-activated manner by calmodulin-dependent protein kinases, especially CaMKKβ.

In all eukaryotes, the β subunits contain a central conserved region that we refer to as the glycogen-binding domain (GBD), which causes AMPK complexes to associate with glycogen in cultured cells and cell-free systems (Hudson et al., 2003; Polekhina et al., 2003). The Pfam database (http://pfam.sanger.ac.uk/) identifies this as an N-isoamylase domain, a noncatalytic domain mainly found in enzymes that metabolize the α1→6 linked branch points in starch and glycogen. The structure of the GBD from rat AMPK-β1 has been determined in the presence of β-cyclodextrin (Polekhina et al., 2005), an unbranched, cyclic α1→4 linked oligosaccharide that does not occur naturally in mammals.

There are many observations correlating glycogen content with the regulation of AMPK, although the underlying molecular mechanisms are poorly understood and there are paradoxes. Prior glycogen loading of skeletal muscle suppresses activation of AMPK by contraction or 5-aminoimidazole-4-carboxamide (AICAR) in rodents (Derave et al., 2000; Wojtaszewski et al., 2002) or exercise in humans (Wojtaszewski et al., 2003). However, AMPK is hyperactivated by exercise in subjects with McArdle disease (who are unable to mobilize glycogen due to an inherited defect in glycogen phosphorylase) despite high glycogen levels (Nielsen et al., 2002). Other paradoxes exist when considering the role of AMPK in regulation of glycogen synthesis. AMPK phosphorylates muscle glycogen synthase (mGS) at site 2 (Ser-7) (Carling and Hardie, 1989). Phosphorylation at this site causes a decrease in activity at low concentrations of the allosteric activator glucose-6-phosphate (Skurat et al., 1994). Phosphorylation of mGS at site 2 occurs in response to AICAR treatment of muscle of wild-type mice, but not AMPK-α2 knockout mice, showing that this site is a physiological target for AMPK (Jorgensen et al., 2004). Because AMPK is activated by exercise, one might expect mGS to be inactivated following exercise. While this is indeed observed in humans with McArdle disease (Nielsen et al., 2002), in normal subjects, mGS is paradoxically found to be dephosphorylated and activated after exercise, an effect that, in mice, requires the G_M_ glycogen-targeting subunit of protein phosphatase-1 (Aschenbach et al., 2001).

There are also interesting but unexplained connections between muscle glycogen content, mGS activity, and insulin-stimulated glucose uptake. Glycogen depletion by electrical stimulation of mouse muscle was shown many years ago (Danforth, 1965) to correlate with an increase in mGS activity indicative of dephosphorylation. In rat muscle, glycogen content appears to have a much greater influence on mGS activity than insulin, with the effect of contraction on mGS being accounted for entirely by glycogen depletion (Nielsen et al., 2001). In rats (Richter et al., 1982) or humans (Mikines et al., 1988), insulin-stimulated glucose uptake and glycogen synthesis is enhanced following a single bout of exercise, and this correlates with the degree of glycogen depletion (Richter et al., 2001).

In this paper, we report that glycogen inhibits purified AMPK in cell-free assays, an effect that is dependent on binding to the GBD and varies according to the branching content of the glycogen. We show that oligosaccharides with single α1→6 branch points are allosteric inhibitors of AMPK that also inhibit phosphorylation and activation by upstream kinases. Our results suggest the concept that the GBD is a regulatory domain that allows AMPK to act as a glycogen sensor in vivo.

## Results

### Identification of Residues within the GBD Required for Glycogen Binding

**Figure 1:**
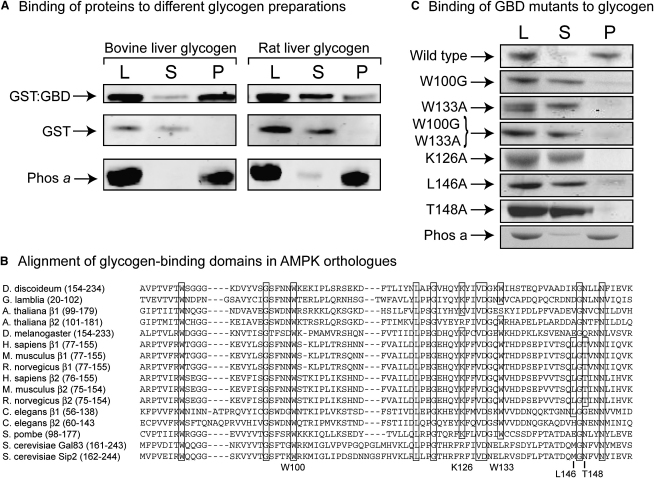
Studies on Binding of Glycogen to Bacterially Expressed β1-GBD (A) Binding of GST:GBD fusion, free GST, and phosphorylase *a* to glycogen. Samples of each protein were incubated with bovine or rat liver glycogen bound to ConA-Sepharose, the Sepharose beads were recovered by centrifugation, and samples of the load (L), supernatant (S), and pellet (P, resuspended in the original volume) were analyzed by SDS-PAGE. (B) Alignment of GBD sequences from various eukaryotes made using ALIGNX. Residues identical in all species are boxed, as are conserved residues in mammalian species directly involved in carbohydrate binding; the latter are identified at the bottom (rat β1 numbering). (C) Binding to glycogen of GST:GBD fusions (wild-type rat β1 or the point mutations shown). The binding assay was as in (A) using bovine liver glycogen, and binding of phosphorylase *a* was analyzed as a positive control (bottom panel).

We first cloned DNA encoding the GBD of the rat AMPK-β1 subunit and expressed it in bacteria as a glutathione S-transferase (GST) fusion. The protein was purified on glutathione-Sepharose to yield a polypeptide with the expected mass of 40 kDa (Figure S1A available online). To examine binding of glycogen to this protein, we developed an assay using Sepharose-bound concanavalin A (ConA), a plant lectin from jack bean that binds glucans via the C3 and C4 hydroxyls of the glucose monomers. Two different preparations of glycogen, a commercial preparation from bovine liver and one made in-house from rat liver, were allowed to bind to ConA-Sepharose. These conjugates were then incubated with the GST:GBD fusion; recovered by centrifugation; and the load, pellets, and supernatants analyzed by SDS-PAGE. Figure 1A shows that the GST-GBD, but not free GST, was recovered almost exclusively in the pellet when bovine liver glycogen was used, indicating efficient binding. The GBD appeared to bind less avidly to the rat liver glycogen, because a larger proportion was found in the supernatant. By contrast, muscle phosphorylase *a*, a protein with a well-defined glycogen-binding site, was recovered almost exclusively in the pellet with both glycogen preparations (Figure 1A).

Figure 1B shows an alignment of the GBD sequences from β subunit isoforms of AMPK orthologs in a variety of different eukaryotic species. A number of residues are conserved throughout mammalian β subunits, including W100, K126, W133, L146, and T148 (rat β1 numbering). The recent crystal structure of the rat β1 GBD in complex with β-cyclodextrin suggested that the side chains of all of these residues form direct interactions with the bound carbohydrate, and mutation of several of them abolished glycogen binding (Polekhina et al., 2003, 2005). To confirm that these residues were involved with glycogen binding, we mutated them to glycine or alanine and tested the ability of the mutant GST-GBD protein to bind glycogen. As expected, all mutations markedly reduced binding of bovine liver glycogen, as did a double-W100G/W133A mutation (Figure 1C).

### Glycogen Preparations Inhibit Purified AMPK with Different Potencies

**Figure 2:**
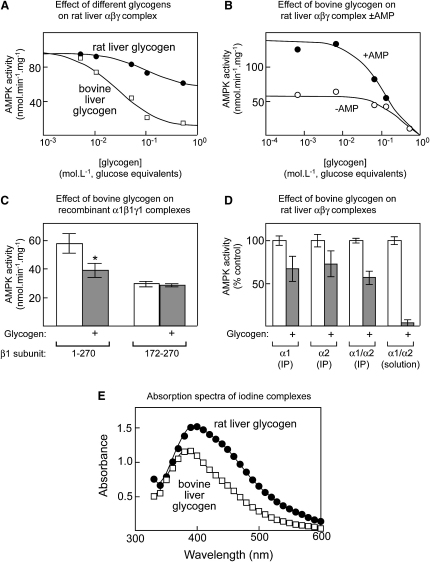
Allosteric Inhibition of AMPK by Different Glycogen Preparations (A) Concentration dependence of inhibition of native rat liver AMPK by preparations of bovine and rat liver glycogen; glycogen concentrations expressed as glucose produced after total hydrolysis. Data were fitted to an IC_50_ equation (see Supplemental Experimental Procedures), and curves were generated using the estimated best-fit parameters. (B) Concentration dependence of inhibition of native rat liver AMPK by bovine liver glycogen in the presence and absence of 200 μM AMP; curves were generated as in (A). (C) Inhibition by bovine liver glycogen of recombinant AMPK complex (*myc*-α1:β1:γ1) containing full-length (1–270) or truncated β1 subunit lacking the GBD (172–270). Complexes were expressed in CCL13 cells and purified by immunoprecipitation prior to assay with or without glycogen (200 mM glucose equivalents). Data are means ± SEM (n = 3). ^∗^Activity different from control without glycogen by t test (p < 0.05). (D) Inhibition by bovine liver glycogen (200 mM glucose equivalents) of purified, native rat liver AMPK (a mixture of α1β1γ1 and α2β1γ1 complexes) that had been recovered by immunoprecipitation (IP) using anti-α1, anti-α2, or a mixture of anti-α1/-α2 antibodies or assayed in solution without immunoprecipitation. Results are expressed as percentages of the activity in controls without glycogen, and data are mean ± SEM (n = 3). (E) Absorption spectra of bovine and rat liver glycogen preparations in complex with iodine.

We next tested the effect of glycogen on the activity of the native AMPK complex purified from rat liver (Hawley et al., 1996). Because they do not have defined structures, for all polysaccharides studied, we express the concentrations in terms of moles of glucose obtained after complete hydrolysis. The bovine liver glycogen inhibited AMPK completely with an IC_50_ (concentration causing half-maximal inhibition) of 30 ± 9 mM glucose equivalents (Figure 2A). By contrast, rat liver glycogen had a much less marked inhibitory effect, causing an extrapolated maximal inhibition of only 44%, with an IC_50_ of 90 ± 16 mM. Although most of the AMPK assays shown in this paper were performed in the presence of 200 μM AMP, the bovine liver glycogen inhibited both in the presence or absence of AMP (Figure 2B), although the inhibition did appear to be somewhat more potent in the presence of AMP.

We were concerned that the more marked inhibition observed with bovine liver glycogen might have been due to the presence of contaminants that inhibited AMPK, but this was not the case. First, the glycogen did not inhibit a bacterially expressed GST fusion of the rat AMPK-α1 kinase domain (data not shown). Second, the inhibition did not occur (Figure 2C) with a recombinant α1β1γ1 complex expressed in human CCL13 cells that had a truncation of residues 1–171 in the β1 subunit that removes the GBD (Hudson et al., 2003). This experiment revealed two additional findings. First, the β subunit truncation reduced the total activity of the complex measured in the absence of glycogen. This has been observed previously (Hudson et al., 2003), although the explanation remains unclear. Second, while the concentration of glycogen used in Figure 2C (200 mM glucose equivalents) produced 90% inhibition of purified native rat liver AMPK (Figures 2A and 2B), inhibition of recombinant AMPK by glycogen (or any of the other carbohydrates tested; see below) was always < 50%. A key difference in the assay conditions used for the native and recombinant AMPK (e.g., Figures 2A and 2C) was that, while the former was performed with the kinase in solution, the latter was performed with the recombinant *myc*-tagged kinase coupled to anti-*myc* antibodies, which was necessary to remove it from the endogenous AMPK in the cells used for expression. To test whether the reduced effect of glycogen was caused by performing the assays in immunoprecipitates, we used rat liver AMPK (an approximately equal mixture of α1β1γ1 and α2β1γ1 complexes) and assayed it either in solution or in resuspended immunoprecipitates made using anti-α1, anti-α2, or a mixture of anti-α1 and anti-α2 antibodies. The results (Figure 2D) show that, when the assays were performed in resuspended immunoprecipitates, the maximal inhibition by glycogen was only 30%–50%, as against > 95% when the assays were performed in solution. Figure 2D also shows that glycogen inhibits the α1β1γ1 and α2β1γ1 complexes purified from rat liver equally well.

We next considered the possibility that the difference in inhibitory potency of the preparations of bovine and rat liver glycogen may have been due to differences in glycogen structure. Given that the GBDs of the AMPK β subunits are related to domains found in enzymes that metabolize α1→6 branch points, an obvious possibility was that the differences were due to differing contents of branching. To examine this, we used a method involving enzymic hydrolysis of the branches followed by determination of the average chain length of the resulting linear α1→4 linked chains. This revealed that the bovine liver glycogen had an average chain length of 13 ± 1 (mean ± SD, n = 3), whereas the rat liver glycogen had an average chain length of 23 ± 3 (mean ± SD, n = 3), indicating a much lower average density of branch points. To confirm this difference using another method, we analyzed the absorption spectra of iodine complexes. The complex between iodine and rat liver glycogen absorbed much more strongly at higher wavelengths than that with the bovine liver glycogen, indicating a lower average degree of branching (Figure 2E).

### Effect of Point Mutations in the GBD on Inhibition by Glycogen

**Figure 3:**
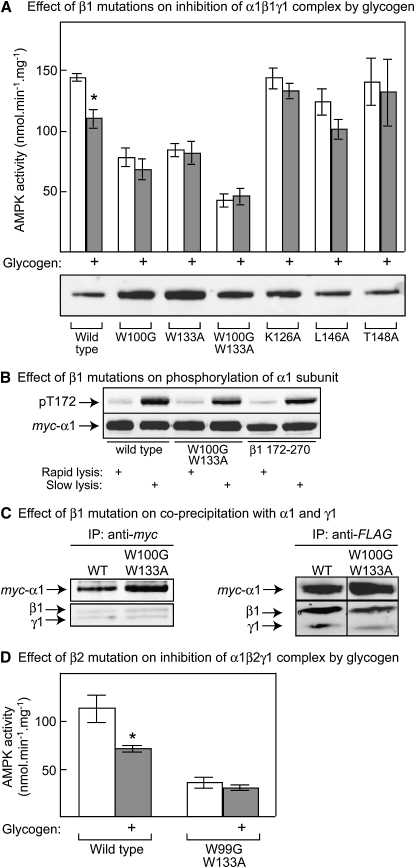
Effect of β Subunit Mutations on Inhibition of Recombinant AMPK Complexes by Glycogen (A) *Myc*-α1, γ1, and β1 with the wild-type sequence or the indicated mutations were expressed in CCL13 cells, immunoprecipitated, and assayed ± bovine liver glycogen (200 mM glucose equivalents); results are mean ± SEM (n = 3). The lower panel shows expression of the α1 subunit assessed by probing blots using anti-*myc* antibody; the activities in the upper panel were corrected for small variations in expression. (B) *Myc*-α1, γ1, and either wild-type β1, a W100G/W133A mutant, or an N-terminally truncated mutant (β1 172–270) were expressed in CCL13 cells, extracted using the rapid or slow lysis procedures, and subject to western blotting using anti-*myc* or anti-pT172 antibodies. (C) *Myc*-α1, γ1, and β1 with the wild-type sequence or with W100G/W133A mutations were expressed in CCL13 cells, immunoprecipitated using anti-*myc* (left) or anti-*FLAG* (right) antibodies, and analyzed by western blotting using anti-*myc* antibodies (top panels) or a mixture of anti-β1 and -γ1 antibodies (bottom panels). (D) *Myc*-α1, γ1, and human β2 with the wild-type sequence or with W99G/W133A mutations were coexpressed, immunoprecipitated, and assayed ± bovine liver glycogen (200 mM glucose equivalents); results are mean ± SEM (n = 3). Activities were corrected for small variations in the observed level of expression as in (A). ^∗^Activity different from control without glycogen by t test (p < 0.05).

To test whether mutations that interfered with the binding of glycogen to the GBD also affected inhibition of AMPK by glycogen, we made mutations in full-length β1 coexpressed with α1 and γ1 in CCL13 cells, isolated the complex by immunoprecipitation via the *myc* tag on α1, and assayed the kinase activity in the presence and absence of glycogen. The activities were adjusted to correct for slight variation in recovery of the α1 subunit assessed by blotting (inset in Figure 3A). All mutations that reduce glycogen binding to the isolated GBD (Figure 1C) also abolished inhibition by bovine liver glycogen. A possible exception was L146A, where some inhibition appeared to remain, although inspection of Figure 1C suggests that this mutation does not completely abolish glycogen binding either. Because all of these assays were conducted in resuspended immunoprecipitates, the degree of inhibition of the wild-type was < 50%, as discussed in the previous section. As observed already with the recombinant heterotrimer containing the truncated β subunit (Figure 2C), all of the mutations except K126A reduced the total activity in the absence of glycogen, albeit to varying extents. This did not appear to be because the mutants were less highly phosphorylated at Thr-172 than the wild-type. In a separate experiment where wild-type β1, truncated β1 (172–270) lacking the GBD, or a W100G/W133A mutant were coexpressed with α1 and γ1, the phosphorylation of Thr-172 on α1 was identical (Figure 3B). This was the case whether the cells were harvested by “rapid lysis” where the cells are lysed in situ using ice-cold buffer containing detergent or “slow lysis” where the cells were harvested by trypsinization and centrifugation prior to lysis. The latter method causes increased phosphorylation due to stresses occurring during cell harvesting. The lack of inhibition by glycogen was also not because the mutant β1 subunits failed to form complexes with α1 and γ1. The W100G/W133A double mutant β1 was recovered in approximately equal amounts with α1 and γ1 whether immunoprecipitated via the *myc* epitope on α1 or via the FLAG epitope on γ1 (Figure 3C).

To test whether binding of glycogen to the GBD caused inhibition of AMPK complexes containing the β2 rather than the β1 isoform, we expressed complexes containing rat α1 and γ1 with human β2, with or without mutation of the two tryptophan residues equivalent to W100 and W133 in β1 (W99G/W133A). The results were very similar to those for β1 in that the wild-type complex was inhibited by glycogen while the double-tryptophan mutation, which reduced the total activity by 70%, also completely abolished the inhibitory effect of glycogen (Figure 3D).

### A Cyclic α1→4 Linked Oligosaccharide Inhibits AMPK, but Linear Oligosaccharides Do Not

**Figure 4:**
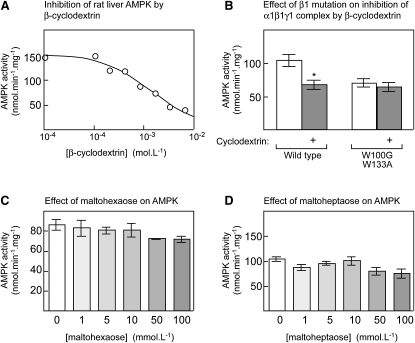
Effect of Unbranched Oligosaccharides on AMPK Activity (A) Inhibition of native rat liver AMPK by β-cyclodextrin. The curve was generated as in Figure 2A. (B) Effect of GBD double mutation on inhibition of recombinant AMPK by 2 mM β-cyclodextrin. *Myc*-α1, γ1, and β1 with the wild-type sequence or with W100G/W133A mutations were coexpressed, immunoprecipitated, and assayed ± β-cyclodextrin (2 mM). The activities were corrected for small variations in the observed level of expression as in Figure 2A. ^∗^Activity different from control without β-cyclodextrin (p < 0.05). (C and D) Effect of maltohexaose (C) and maltoheptaose (D) on AMPK activity. Native rat liver AMPK was assayed in the presence and absence of the indicated concentrations of oligosaccharide. Results in (B) through (D) are mean ± SEM (n = 3).

The different results obtained with bovine and rat liver glycogen illustrate a major problem in studying glycogen as a regulatory molecule, in that the polysaccharide does not have a defined structure and varies in both size and degree of branching. Polekhina et al. (2003, 2005) have previously shown that β-cyclodextrin, a cyclic α1→4 linked oligosaccharide of seven glucose units, can compete with glycogen for binding to the rat β1 GBD. Therefore, we tested whether β-cyclodextrin also caused inhibition. Figure 4A shows that this was indeed the case with a half-maximal effect at 1.6 mM, which is close to the K_D_ of 0.3 mM measured for binding of β-cyclodextrin to the bacterially expressed rat β1 GBD by Koay et al. (2007). This inhibition was dependent on binding to the GBD because it was abolished by the double-tryptophan mutation (W100G/W133A) in β1 (Figure 4B).

Polekhina et al. (2005) also found that linear α1→4 linked oligosaccharides with more than five glucose units, i.e., maltohexaose and maltoheptaose, could displace the β1 GBD from glycogen at concentrations greater than 1 mM. Maltoheptaose is equivalent to β-cyclodextrin but with a break in the chain so that it has free reducing and nonreducing ends. Surprisingly, these linear oligosaccharides produced almost no inhibition of native rat liver AMPK even at concentrations up to 100 mM (Figures 4C and 4D), although we did confirm that they could bind the GBD (data not shown). Using an NMR method, Koay et al. (2007) estimated K_D_ values of 0.7 and 0.4 mM for binding of maltohexaose and maltoheptaose to the bacterially expressed rat β1 GBD. Thus, these linear oligosaccharides do not inhibit AMPK even at concentrations where binding should be saturated.

### Isolation of a Fragment of Glycogen that Binds to the GBD and Inhibits AMPK

**Figure 5:**
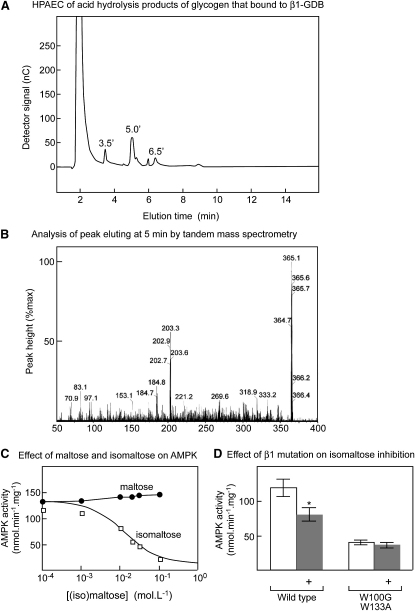
Isolation of Isomaltose as an Inhibitor of AMPK (A) Bovine liver glycogen was subjected to partial acid hydrolysis and the hydrolysate passed through a glutathione Sepharose column to which the GST:GBD fusion had been prebound. The column was eluted with propionic acid and bound oligosaccharides analyzed by HPAEC. The peak eluting at 5 min was the only one found to contain carbohydrate. (B) Analysis of the peak eluting at 5 min by tandem ES-MS using collision-induced dissociation. Oligosaccharide fragments are observed as adducts with Na^+^ ions, increasing their mass by 23. (C) Effect of maltose and isomaltose on AMPK activity. The curve for isomaltose was generated using the best-fit parameters as in Figure 2A. (D) Effect of GBD double mutation on inhibition of recombinant AMPK by 20 mM isomaltose; results are mean ± SEM (n = 3). ^∗^Activity different from control without isomaltose by t test (p < 0.05).

In an attempt to establish the chemical nature of the regions of glycogen that bind to the GBD and cause inhibition of the AMPK complex, we carried out partial acid hydrolysis of bovine liver glycogen and utilized the β1 GBD as an affinity matrix to purify hydrolysis products that bound to the GBD. The hydrolysis conditions were chosen to yield a range of oligosaccharides containing up to six glucose units, as detected by high-performance anion exchange chromatography (HPAEC). The hydrolysate was passed down a glutathione Sepharose column to which the GST-GBD had been prebound, and bound oligosaccharides eluted with propionic acid. The eluted oligosaccharides were analyzed by HPAEC (Figure 5A). A large peak at 1.7 min was shown to be an artifact caused by propionic acid, while three peaks at 3.5, 5.0, and 6.5 min were analyzed by electrospray ionization-mass spectrometry. The peaks at 3.5 and 6.5 min did not appear to contain carbohydrate, but the peak at 5 min yielded a mass of 365, consistent with a disaccharide. That this disaccharide contained glucose was confirmed by collision-induced dissociation and tandem mass spectrometry (Figure 5B); the product ion spectrum contained a daughter peak at 203, corresponding to the mass of glucose plus one Na^+^ ion. The only disaccharides expected from partial acid hydrolysis of glycogen are maltose (α1→4 linked) and isomaltose (α1→6 linked). Attempts to identify the disaccharide by chemical linkage analysis were not successful. However, an isomaltose standard was found to elute from the HPAEC column at 5.1 min, whereas maltose eluted at 10.1 min. These results suggested that the disaccharide that was derived from glycogen and that bound to the β1-GBD was isomaltose.

Interestingly, isomaltose inhibited native rat liver AMPK with a half-maximal effect at 16 mM, whereas maltose did not cause any inhibition at concentrations up to 100 mM (Figure 5C). As with bovine liver glycogen, isomaltose did not inhibit a T172D mutant of the α1 kinase domain expressed in bacteria (data not shown). As observed with other inhibitory glucans, isomaltose inhibited wild-type recombinant α1β1γ1 complexes, but not complexes containing a double-tryptophan mutation in β1 (Figure 5D).

### Effect of Synthetic Branched Oligosaccharides on AMPK Activity

**Figure 6:**
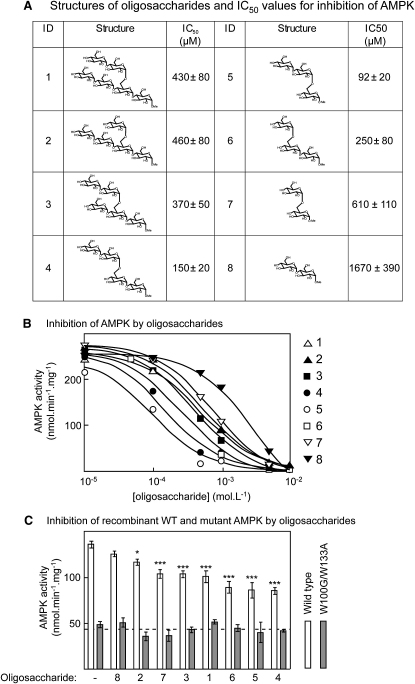
Inhibition of Rat Liver AMPK by Synthetic Branched Oligosaccharides (A) Table showing the identification (ID) number, structure, and estimated IC_50_ ± SEM. (B) Inhibition of purified rat liver AMPK by oligosaccharides. These data were used to generate the IC_50_ values shown in (A), and the curves were drawn using the best-fit parameters. A key to the ID of each oligosaccharide is shown on the right. (C) Inhibition of recombinant wild-type and W100G/W133A mutant AMPK (α1β1γ1 complex) by synthetic oligosaccharides, numbered as in (A) and (B). The data are presented with the oligosaccharides in order of increasing potency from left to right; results are mean ± SEM (n = 3). Significant differences ± oligosaccharide for the wild-type by two-way ANOVA. ^∗^p < 0.05; ^∗∗∗^p < 0.001.

To test whether oligosaccharides larger than isomaltose with an α1→6 linkage would be more potent inhibitors, we chemically synthesized a series of α1→4 linked oligosaccharides, containing from three to six glucose units, with a single α1→6 linkage (Figure 6A). Because oligosaccharides with a free reducing end exist in solution as mixtures of α and β anomers, these oligosaccharides were synthesized with a methoxy group at carbon 1 of the glucose at the reducing end, in the α configuration. For comparison, we also synthesized methyl α-maltoside and methyl α-isomaltoside.

These oligosaccharides were tested for their ability to inhibit AMPK (Figure 6B; the structures and estimated IC_50_ values are shown in Figure 6A). Surprisingly, whereas maltose with a free reducing group did not inhibit AMPK, methyl α-maltoside did inhibit, with an IC_50_ of 1.7 mM. Inhibition was also more potent with methyl α-isomaltoside (IC_50_ = 0.6 mM) than with isomaltose (IC_50_ = 16 mM). Intriguingly, α1→4 linked glucose oligosaccharides containing from three to six glucose units and a single α1→6 linkage were more potent inhibitors, all with IC_50_ values of less than 0.5 mM. The most potent was a trisaccharide containing two glucose units on the secondary (branch) chain and a single α-methyl glucoside on the primary chain (Glcα1→4Glcα1→6Glcα-OMe), which had an IC_50_ of 90 μM.

To confirm that the inhibition by these oligosaccharides was dependent on binding to the GBD, we tested their effects on recombinant α1β1γ1 complexes expressed in CCL13 cells. As expected, they all inhibited wild-type AMPK, although, at the single concentration used (2 mM), the inhibition by methyl α-maltoside was not significant. However, none of them inhibited recombinant complexes containing the double-W100G/W133A mutation in β1 (Figure 6C). As observed in other experiments, there was a decrease of about 70% in the activity of the double mutant in the absence of oligosaccharide.

### Effect of Branched Oligosaccharides on Phosphorylation/Dephosphorylation of AMPK

**Figure 7:**
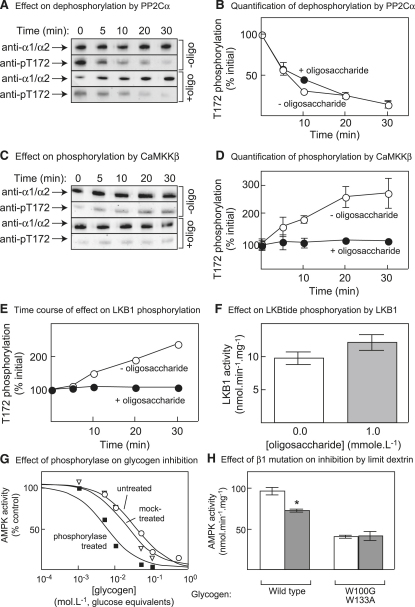
Effect of Oligosaccharide Number 5, See Figure 6, on Dephosphorylation of AMPK by PP2Cα and on Phosphorylation by CaMKKβ and LKB1 (A) Effect of oligosaccharide on dephosphorylation by PP2Cα. Rat liver AMPK was incubated with PP2Cα in the presence and absence of 1 mM oligosaccharide; at various times, levels of total α subunit and phosphorylation on Thr-172 were assessed by western blotting. (B) Quantification of results as in (A); means ± SEM of two experiments. (C) Effect of oligosaccharide on phosphorylation by CaMKKβ. Rat liver AMPK was dephosphorylated using PP1, okadaic acid was added to inhibit the phosphatase, and the protein was incubated with MgATP and CaMKKβ in the presence and absence of 1 mM oligosaccharide. At various times, levels of total α subunit and of phosphorylation on Thr-172 were assessed by western blotting. (D) Quantification of results as in (C); means ± SEM (n = 2). (E) Time course of effect of oligosaccharide on phosphorylation by LKB1, performed as in (C) but with LKB1 in place of CaMKKβ. (F) Effect of oligosaccharide on phosphorylation of a synthetic peptide substrate of LKB1 (mean ± SD; n = 2). (G) Inhibition of AMPK by bovine liver glycogen, a phosphorylase limit dextrin, and a mock-treated sample. IC_50_ values were determined as in Figure 2A and curves generated from the best-fit parameters. (H) Effect of limit dextrin on recombinant α1β1γ1 complex with wild-type and W100G/W133A mutant β1. Results are mean ± SEM (n = 3). ^∗^Activity different from control without limit dextrin (p < 0.05).

The inhibitory effects observed to this point represent allosteric inhibition, i.e., direct effects on the activity of the AMPK kinase domain of carbohydrate binding at a different site, i.e., the GBD on the β subunit. It was important to establish whether carbohydrates might also affect phosphorylation and dephosphorylation by upstream kinases and phosphatases. Figures 7A and 7B show that the most potent inhibitory branched oligosaccharide (Glcα1→4Glcα1→6Glcα-OMe) had no significant effect on dephosphorylation of purified rat liver AMPK by protein phosphatase-2Cα (PPM1A). Similar negative results were obtained using the catalytic subunit of protein phosphatase-1 (data not shown). However, the trisaccharide did cause a marked inhibition of phosphorylation of Thr-172 by CaMKKβ (Figures 7C and 7D) and LKB1 (Figure 7E). To confirm that the latter was not an effect on LKB1, we tested the effect of the oligosaccharide on phosphorylation of a synthetic peptide substrate, i.e., LKBtide (Lizcano et al., 2004). There was no significant inhibition at 1 mM oligosaccharide (Figure 7F), when inhibition of phosphorylation of the rat liver αβγ complex was essentially complete (Figure 7E).

### Increased Inhibition by a Phosphorylase Limit Dextrin

Neither isomaltose nor any of the synthetic branched oligosaccharides are likely to occur naturally in vivo, although they can be considered as mimics of the branch points in glycogen. To examine the effects of the branch points in glycogen itself, we studied the effects of phosphorylase digestion. Phosphorylase degrades the outer chains of glycogen sequentially by phosphorolysis but stops when close to the branch points (Walker and Whelan, 1960). We reasoned that, if we digested glycogen exhaustively with phosphorylase in the absence of debranching enzyme, we would generate a limit dextrin in which the branch points would be exposed on the surface. By contrast, in undigested glycogen, the branch points would be buried beneath the outer chains and may be less accessible to AMPK. Our prediction was that the limit dextrin would be a more potent inhibitor than the parent glycogen.

During initial trials of this approach, we found that commercial preparations of phosphorylase are contaminated with debranching enzyme, which had to be removed by further purification. Using highly purified phosphorylase, the release of glucose as glucose-1-phosphate from bovine liver glycogen reached a plateau corresponding to 28% release of the total glucose in the glycogen. This is the expected amount because about 30% of the glucose in an undegraded glycogen molecule is expected to be in the outer tier, irrespective of the number of tiers (Melendez-Hevia et al., 1993). To prepare the limit dextrin, we incubated the glycogen with the cosubstrate phosphate with or without phosphorylase and also kept a sample of the untreated parent glycogen. Since the removal of the unbranched outer chains by phosphorylase treatment would increase the average degree of branching, this was assessed by measuring the absorption spectrum of the complex between the polysaccharide product and iodine. The λmax values of the complexes with untreated glycogen, mock-treated glycogen, and phosphorylase-treated glycogen were 397, 372, and 350 nm, respectively. This indicates that, while the phosphorylase treatment did remove the outer branches and, thus, increase the average degree of branching, there was also some increase in branching content in the mock-treated sample, presumably because of trace contamination of the commercial preparation of bovine liver glycogen with active phosphorylase.

As predicted (Figure 7G), the phosphorylase treatment increased the potency of inhibition of rat liver AMPK, with the IC_50_ decreasing from 38 to 5 mM (glucose equivalents). The mock-treated sample yielded inhibition of intermediate potency (IC_50_ = 21 mM), consistent with the idea that some phosphorolysis may have occurred. As with other inhibitory carbohydrates, the inhibition of a recombinant α1β1γ1 complex by the phosphorylase-treated limit dextrin was abolished by the W100G/W133A mutation (Figure 7H).

## Discussion

The results in this paper reveal that the glycogen-binding domain on the β subunits of AMPK, rather than just localizing AMPK on glycogen, is also a regulatory domain that allosterically inhibits the kinase activity (and inhibits phosphorylation by upstream kinases) when glycogen is bound, thus allowing AMPK to act as a glycogen sensor. We were initially concerned that inhibition of AMPK by the commercial bovine liver glycogen might be explained by contamination with molecules that interfered in the assay. However, the glycogen preparation did not inhibit the isolated AMPK-α1 kinase domain, showing that it was not a nonspecific inhibitor of the catalytic activity. Moreover, inhibition was dependent on binding of glycogen to the GBD, because it was not observed with recombinant α1β1γ1 complexes containing a truncated β subunit lacking the domain or containing point mutations in the β subunit that disrupt glycogen binding.

A major problem with the study of glycogen as a regulatory molecule is that it does not have a defined structure, so different preparations may behave quite differently. This is illustrated by the dramatic difference in inhibitory effect between the preparations of bovine and rat liver glycogen. This may also account for the findings of Polekhina et al. (2003), who reported that glycogen did not inhibit purified rat liver AMPK, and for the failure of Parker et al. (2007) to find any AMPK associated with glycogen purified from rat liver. Parker et al. (2007) purified glycogen using a procedure involving sucrose gradient centrifugation and size exclusion chromatography, and another possibility is that AMPK dissociated during this procedure.

A consistent finding throughout this study was that, while native rat liver AMPK (a mixture of the α1β1γ1 and α2β1γ1 complexes [Woods et al., 1996]) was completely inhibited by bovine liver glycogen and branched oligosaccharides, recombinant AMPK complexes expressed in human cells and assayed in immunoprecipitates were inhibited to a much lower extent, usually only by 30%–50%. However, this was due to carrying out the assays in immunoprecipitates rather than in solution, because a similar incomplete degree of inhibition was obtained in resuspended immunoprecipitates obtained from preparations of native rat liver AMPK using anti-α1 or -α2 antibodies. These experiments also revealed that native complexes from rat liver containing either the α1 or the α2 isoform were both susceptible to inhibition by glycogen.

Why did the preparations of bovine and rat liver glycogen differ markedly in their ability to inhibit AMPK? We suspected that this might be due to different degrees of branching. In support of this, the average branching content of the rat liver glycogen was found to be much less than that of the bovine liver glycogen, a result confirmed by examining the absorption spectra of the iodine complexes. Most published estimates of the average chain length of mammalian glycogen suggest that it lies between 12 and 18 glucose units (Illingworth et al., 1952), in line with our value of 13 ± 1 for bovine liver glycogen but making our value of 23 ± 3 for the rat liver glycogen seem high. However, the latter was not purified by the traditional extraction in hot alkali but by a method designed for purification of glycogenin involving successive extractions with cold trichloroacetic acid, LiBr, and SDS (Smythe et al., 1989). It is possible that these two methods select out subfractions of glycogen with different degrees of branching.

The idea that it is the branch points in glycogen that bind with high affinity to the GBD and cause inhibition of AMPK was supported by our findings that, following partial acid hydrolysis of bovine liver glycogen, isomaltose was recovered by binding to the immobilized β1 GBD. Isomaltose was found to inhibit AMPK, whereas maltose did not. We also found that maltohexaose and maltoheptaose (containing six and seven glucose units all α1→4 linked) did not inhibit AMPK significantly, although they do bind to the GBD (Koay et al., 2007).

Maltose, isomaltose, maltohexaose, and maltoheptaose all have a free reducing end that can interconvert via the open chain form to give a mixture of α- and β-anomers, although the β-anomer is thought to predominate. We synthesized derivatives of maltose and isomaltose with an α-methoxy group at C1 of the reducing end and found that (unlike maltose itself) methyl α-maltoside did inhibit AMPK, while methyl α-isomaltoside was a much more potent inhibitor than isomaltose. In addition, β-cyclodextrin (a cyclized form of maltoheptaose that lacks a free reducing end) inhibited AMPK, whereas maltoheptaose did not. These results suggest that the presence of a free reducing end somehow abolishes the inhibition of AMPK by oligosaccharides, possibly due to the presence of the β-anomer. Although the molecular explanation for this phenomenon remains unclear, polysaccharides or oligosaccharides with free reducing ends are unlikely to occur in vivo.

We suspected that the true physiological ligands for the GBDs of AMPK might be the branch points in glycogen, which isomaltose was mimicking. To generate better models for such branch points, we synthesized a series of oligosaccharides containing from three to six α1→4 linked glucose units with a single α1→6 linkage and an α-methoxy group at the reducing end. These small, branched oligosaccharides were more potent inhibitors of AMPK than methyl α-isomaltoside. The most potent was a trisaccharide (Glcα1→4Glcα1→6Glcα-OMe) with two glucose units on the branched side chain, which had an IC_50_ 9-fold lower than methyl α-isomaltoside and almost 20-fold lower than methyl α-maltoside. This may represent the smallest ligand that makes all possible favorable contacts with the binding site on the GBD.

A molecule of glycogen is thought to have a theoretical maximum of 12 tiers of branching, because, above this limit, the outer chains may become so tightly packed that no further branching can be accommodated (Melendez-Hevia et al., 1993). If this view of glycogen structure is correct, it would imply that AMPK would only be able to bind to the nonreducing ends at the surface of a full-size glycogen molecule and may not be able to gain access to internal branch points. However, as phosphorylase degrades the outer chains of glycogen, it would expose the outer tier of branch points. Phosphorylase stops degrading the outer chains when four glucose units remain on the side chain (Walker and Whelan, 1960) and cannot proceed further until the branches are resolved by debranching enzyme. Therefore, we suspected that a limit dextrin made by exhaustive digestion of bovine liver glycogen with phosphorylase might be a more potent inhibitor of AMPK than the parent glycogen. This was, indeed, the case (Figure 7G). Thus, removal of the outer chains of glycogen by phosphorylase markedly increases the potency of inhibition of AMPK.

Our results confirm the idea that the GBDs of the AMPK β subunits represent a regulatory domain that inhibits the kinase activity and suggest that branch points within glycogen are the true inhibitory ligands. Although much further work is required to establish the physiological role of this phenomenon, we suggest the following working hypothesis. We propose that a significant proportion of AMPK, especially in muscle containing a high glycogen content, may be bound to the nonreducing ends at the surface of glycogen, partially sequestering AMPK away from other downstream targets. This could account for the reduced apparent activation of AMPK in response to AICAR (Wojtaszewski et al., 2002) or contraction (Derave et al., 2000; Wojtaszewski et al., 2003) when muscle is in a glycogen-loaded state. However, because the densely packed outer chains may prevent access to most of the internal branch points, AMPK bound to glycogen under these conditions would not be in an inhibited state and would phosphorylate mGS at site 2, providing a feedback inhibition of the further extension of the outer glycogen chains. If muscle contraction then commenced and phosphorylase was activated, it would expose the outermost tier of branch points (assuming that debranching enzyme did not immediately resolve them), which would now inhibit AMPK. AMPK would no longer phosphorylate mGS, allowing the latter to become dephosphorylated and activated, paving the way for rapid glycogen resynthesis once contraction ceased.

Our hypothesis can explain many of the observations and paradoxes discussed in the Introduction. For example, it can explain why mGS is less active due to phosphorylation at site 2 in glycogen-loaded muscle (Jorgensen et al., 2004; Lai et al., 2007). It also explains why, following glycogen depletion caused by electrical stimulation in rat muscle (Danforth, 1965; Nielsen et al., 2001) or exercise in human muscle (Wojtaszewski et al., 2001), mGS is found to be in an active, dephosphorylated state. The AMPK bound to glycogen would be inhibited under these conditions, allowing net dephosphorylation of mGS by the glycogen-bound form of protein phosphate-1 (Aschenbach et al., 2001).

As well as causing allosteric inhibition, the trisaccharide Glcα1→4Glcα1→6Glcα-methyl inhibited phosphorylation by upstream kinases, but not dephosphorylation by PP2Cα or protein phosphatase-1. This contrasts with the effect of the well-established allosteric activator of AMPK, i.e., AMP, which inhibits dephosphorylation without affecting phosphorylation (Davies et al., 1995; Suter et al., 2006; Sanders et al., 2007). Inhibition of Thr-172 phosphorylation by glycogen may account for findings that AMPK is more highly phosphorylated at this site in rat muscle that has a low glycogen content (Jorgensen et al., 2004). However, it seems likely that the level of Thr-172 phosphorylation would rapidly revert to normal once AMPK had dissociated from glycogen, i.e., that the inhibitory effect of glycogen would only be experienced by those AMPK targets that are resident in the glycogen particle, like mGS.

Our hypothesis may also help to explain the increase in insulin-stimulated glucose uptake following a single bout of exercise (Richter et al., 1982; Mikines et al., 1988), which correlates with the extent of glycogen depletion (Richter et al., 2001). Removal of the outer tier of glycogen by phosphorylase releases about 30% of the available glucose and halves the number of nonreducing ends (Melendez-Hevia et al., 1993). Thus, even a modest reduction in glycogen content might cause release of significant quantities of AMPK from the polysaccharide so that more of the kinase becomes available to phosphorylate targets involved in glucose uptake and/or insulin signaling.

In conclusion, our results suggest the hypothesis that the AMPK complex can sense not only the immediate availability of cellular energy in the form of AMP and ATP but also the availability of medium-term energy reserves in the form of glycogen.

## Experimental Procedures

Experimental procedures used in this study are presented in the Supplemental Data.

## Supplemental Data

Document S1. Supplemental Experimental Procedures, One Figure, and One Table
