# Supplementary material for: The Glycogen-Binding Domain on the AMPK β Subunit Allows the Kinase to Act as a Glycogen Sensor
Source: Cell Metab. 2009 Jan 7;9(1):23–34. doi: 10.1016/j.cmet.2008.11.008 (PMC2642990; doi:10.1016/j.cmet.2008.11.008)
Supplement: Document S1. Supplemental Experimental Procedures, One Figure, and One Table [file mmc1.pdf]

Cell Metabolism, *Volume 9*

## Supplemental Data

# The Glycogen-Binding Domain on the AMPK β Subunit Allows the Kinase to Act as a Glycogen Sensor

Andrew McBride, Stephanos Ghilagaber, Andrei Nikolaev, and D. Grahame Hardie

**Figure S1: Purification of GST-GBD Fusion from Bacteria**

The rat β1 GBD (residues 70-172 was cloned into pGEX6P2, expressed in *Escherichia coli* (BL21) and purified on a glutathione-Sepharose column. The picture shows a Coomassie Blue-stained gel following analysis by SDS-PAGE of molecular mass markers (M), the cell lysate (L), the flow-through (FT), two fractions of the wash (W1 and W2) and fractions of the glutathione eluate (F4-F8). The GST-GBD migrated with a mobility corresponding to 40 kDa as expected.

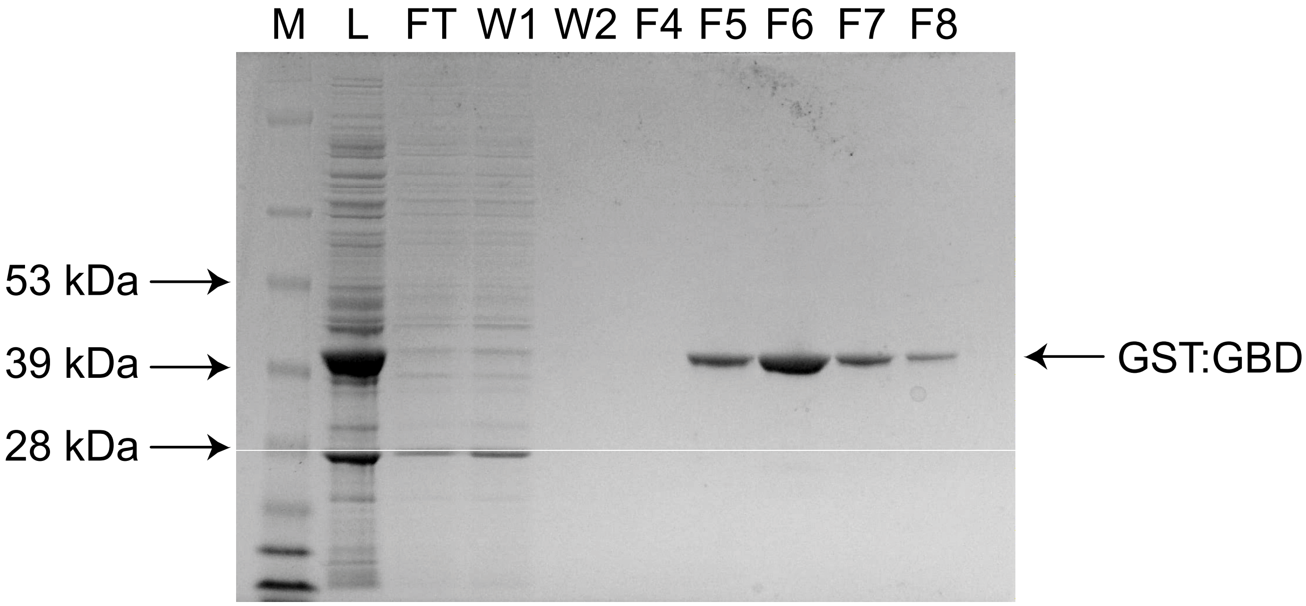

**Table S1: Oligonucleotide Primers Used in this Project**

| Primer ID | Orientation | Sequence |
|---|---|---|
| β1-GBD insert (70-172) | sense | 5′-ctagaattcacgacctcgaggtgaatgag-3′ |
| | antisense | 5′-ccagtcgacggacagctcagatacatcgg-3′ |
| β1 mutagenesis (W100G) | sense | 5′-tccttcaacaacgggagcaaattgcccctcactag-3′ |
| | antisense | 5′-ccgttaaacgagggcaacaacttcctaggtctgttc-3′ |
| β1 mutagenesis (W133A) | sense | 5′-gtggatggccaggcgacccacgatccttccgag-3′ |
| | antisense | 5′-aaggatcgtgggtcgcctggccatccacaaagaac-3′ |
| β1 mutagenesis (K126A) | sense | 5′-ggagagcatcagtacgcgttctttgtggatggccag-3′ |
| | antisense | 5′-catccacaaagaacgcgtactgatgctctccttccgg-3′ |
| β1 mutagenesis (L146A) | sense | 5′-ccaatagtaaccagccaggctggcacag-3′ |
| | antisense | 5′-gaatgatgttgttaactgtgccagcctggctgg-3′ |
| β1 mutagenesis (T148A) | sense | 5′-accagccagcttggcgcagttaacaacatc-3′ |
| | antisense | 5′-tgatgttgttaactgcgccaagctggctgg-3′ |
| β2 mutagenesis (W99G) | sense | 5′-ttcaacaatgggagcaccaagattccac-3′ |
| | antisense | 5′-aatcttggtgctcccattgttgaaagaccc-3′ |
| β2 mutagenesis (W133A) | sense | 5′-gatggacaggcggttcatgatccatcag-3′ |
| | antisense | 5′-atcatgaaccgcctgtccatccacaaag-3′ |

## Supplemental Experimental Procedures

### *Materials*

Unless stated otherwise below, biochemical reagents including glycogen (bovine liver type IX), maltose, isomaltose, maltohexaose, maltoheptaose, β-cyclodextrin, glycogen phosphorylase *a*, glucose oxidase, horseradish peroxidase, amyloglucosidase (from *Aspergillus niger*) and lysozyme were from Sigma-Aldrich, Gillingham, UK. Isopropyl β-D-1-thiogalactopyranoside (IPTG), dithiothreitol (DTT) and molecular biology grade agarose were from Melford Labs, Ipswich, UK. SeeBlue Plus2 molecular mass standards, NuPAGE gel systems, Dulbecco's Modified Eagle's Medium (DMEM) plus GlutaMAX1, RPMI-1640 medium, fetal bovine serum (FBS), trypsin/EDTA solution, penicillin/streptomycin, One Shot TOP10F' and BL21(DE3) chemically competent *E. coli* cells were from Invitrogen, Paisley, UK. Nitrocellulose and bovine serum albumin standards were from BioRad Laboratories, Hemel Hempstead, UK. Reduced glutathione and Complete EDTA-free protease

inhibitor cocktail were from Roche, Burgess Hill, UK. DNA markers, restriction endonucleases and T4 DNA ligase were from New England Biolabs, Hitchin, UK. Pfu DNA polymerase and the QuikChange directed mutagenesis kit was from Stratagene, Amsterdam, The Netherlands. Qiaprep spin mini-prep kits, Plasmid maxi Hi-speed kits and PCR purification kits were from Qiagen, Crawley, UK. Oligonucleotides were synthesized by Sigma-Genosys, Cambridge, UK. [$\gamma$-$^{32}$P]ATP, ConA-Sepharose, FF-trap Glutathione-Sepharose columns (5 ml), CNBr-activated Sepharose 4 Fast Flow, DEAE-Sepharose, protein G-Sepharose, aminobutyl agarose and Optiscint Hisafe scintillation fluid were from GE Healthcare, Little Chalfont, UK. P81 paper and 3MM filter paper were from Whatman International, Maidstone, UK. Isoamylase was from MP Biomedicals, Cambridge, UK.

### *Antibodies*

Anti-$\alpha$1 and -$\alpha$2 antibodies have been described (Woods et al., 1996). Anti-*myc* (#2276) and anti-AMPK pT172 (#2532) antibodies were from Cell Signaling Technologies, New England Biolabs, Hitchin, UK. Anti-GST antibodies were purified as a by-product of the production of antibodies against GST-tagged LKB1. They were recovered from an anti-GST-LKB1 antiserum using an immobilized GST column. IR dye-labelled secondary antibodies (anti-sheep, -mouse and -rabbit) were from Li-COR Biosciences UK, Cambridge, UK. The anti-*myc* antibodies from Cell Signaling were used to probe immunoblots; for immunoprecipitation we used antibodies prepared in house from the growth medium of Myc9E10 hybridoma cells (ATCC, LGC PromoChem, Teddington, UK). The cells were cultured in RPMI-1640 medium supplemented with 10% FBS, at 37$^o$C, 5% $CO_2$. Cells were removed by centrifugation (2000 rpm; 15 min). $(NH_4)_2SO_4$ was slowly added to the supernatant with gently stirring at 4$^o$C to 50% saturation. The suspension was stirred for a further 30 min prior to collection of the precipitate (22,000g; 30 min). The precipitate was resuspended in 10% of the original volume in phosphate-buffered saline (PBS), and dialysed against PBS for 24 hr with frequent changes. To couple the antibody to protein G-Sepharose beads, the beads were pre-washed in 3 x 3 vols of IP buffer (50 mM Tris/HCl, pH 7.3, 1 mM EGTA, 1 mM EDTA, 50 mM NaCl, 5 mM Na pyrophosphate, 50 mM NaF, 2 mM DTT, 0.1 mM PMSF, 2 mM benzamidine, 1% (v/v) Triton X-100), prior to

incubation with 10 vols of dialysed anti-*myc* antibody at 4$^o$C with gentle mixing for 2 hr. Beads were then washed in 4 x 3 vols of IP buffer and stored at 4$^o$C.

### *Plasmids*

pcDNA3 plasmids encoding myc-tagged $\alpha$1(rat), $\beta$1(rat), $\beta$2(human) and $\gamma$1(rat) were gifts from Dr. David Carling (Woods et al., 1996; Thornton et al., 1998). pGEX6P2 was from GE Healthcare, Little Chalfont, UK.

### *Purification of Native Rat Liver, AMPK, Recombinant AMPK, and AMPK Assays*

AMPK was purified from rat liver as far as the gel filtration step (Hawley et al., 1996). The kinase was assayed using the *SAMS* peptide (Davies et al., 1989). $IC_{50}$ values for inhibition were estimated by fitting data using GraphPad Prism to the equation: Activity = Residual + Maximum - (Maximum × [Inhibitor]/($IC_{50}$+[Inhibitor])) where Residual is the activity in the presence of maximal inhibition, Maximum is the maximal inhibition and [inhibitor] is the concentration of the inhibitory molecule. For recombinant AMPK, constructs encoding *myc*-tagged $\alpha$1, $\beta$1 and $\gamma$1 subunits of AMPK were co-transfected into CCL13 cells using modifications of previous methods (Woods et al., 1996). DNA (10 µg) was dissolved in sterile water (450 µl) to which 50 µl of 2.5 M $CaCl_2$ was added, and the solution vortexed for 1 min. Finally, 500 µl of 2 × BES buffer (50 mM N,N-bis(2-hydroxyethyl)-2-aminoethanesulfonic acid, 280 mM NaCl, 1.5 mM $Na_2HPO_4$, pH 6.96) was added, the solution vortexed for 1 min, and allowed to stand at room temp for 40 min. The solution was added drop-wise to CCL13 cells (60-70% confluent) in 10 ml DMEM medium supplemented with 10% FBS. Following transfection, the cells were incubated overnight at 37°C in 5% $CO_2$. The medium was aspirated, fresh medium (10 ml) added and the cells incubated for a further 24 hr. Cells were subject to rapid or slow lysis (Scott et al., 2004) (rapid lysis was used unless stated otherwise) and AMPK assayed in immunoprecipitates as described previously (Hardie et al., 2000).

### *Synthetic Oligosaccharides*

Detailed methods for the synthesis of the mixed α1→4, α1→6 oligosaccharides, methyl α-maltoside and methyl α-isomaltoside will be presented elsewhere.

### *Purification of Glycogen from Rat Liver*

Glycogen was purified from the livers of 11 rats using a protocol modified from Smythe et al (1989). Briefly, the livers were homogenised in 2.5 vols of 10% (w/v) trichloroacetic acid and cell debris removed by centrifugation (5,000 x g; 40 min). LiBr was added to the supernatant (1 mM), glycogen precipitated in 3 vols ethanol and collected by centrifugation (4,000 x g; 30 min). The pellet was dissolved in 100 ml water. Lipids were extracted using chloroform/methanol (3:1) prior to reprecipitation of glycogen in 3 vols ethanol as before. The precipitate was dissolved in 140 ml of 1% (w/v) Na dodecyl sulphate. This solution was centrifuged (250,000 x g; 2 hr) and the pellet dissolved in water prior to re-precipitation of glycogen in 3 vols of ethanol as before. The precipitate was dissolved in 50 mM $NH_4HCO_3$, pH 8.1. This solution was applied to a DEAE-Sepharose column (3 x 3 cm) equilibrated in the same buffer. The flow-through was collected and glycogen precipitated with 3 vols of ethanol as before, before being dissolved in water and stored in aliquots at -20°C.

### *Quantification of Glucose Content of Glycogen and Phosphorylase Limit Dextrin*

Glycogen or limit dextrin in a final volume of 100 μl was digested with 100 μl of 5 U/ml amyloglucosidase in 0.2 M Na acetate, pH 4.8, at 40$^o$C for 2 hr. Sample (40 μl) were transferred in duplicate to 96 well plates and neutralised with 10 μl of 250 mM NaOH. The volume of the neutralised sample was adjusted to 100 μl with 0.1 M phosphate buffer pH 7.0. To each well, 100 μl of assay mix (1 U/ml glucose oxidase, 0.1 U/ml horseradish peroxidase and 750 μM ABTS (2,2'-azino-bis(3-ethylbenzthiszoline-6-sulfonic acid) in 0.1 M phosphate buffer pH 7.0) was added and incubation continued in darkness for 10 min. The absorbance at 405 nm was then measured using a Fluostar Optima Plate Reader (BMG Lab Technologies, Aylesbury, UK). A standard curve was constructed using a range (0-4 μg) of glucose.

### *Determination of the Degree of Glycogen Branching*

The degree of branching of a known mass of glycogen was determined by comparison of the number of reducing termini before and after incubation with isoamylase (Takeda et al., 1993). In some experiments, branching was estimated by determining the wavelength of maximum absorption (λmax) of the glycogen-iodine complex (Krisman, 1962). To 100 ml of 0.5 mg/ml glycogen, 650 ml of Krisman's reagent (6 mM KI, 0.4 mM $I_2$ in saturated $CaCl_2$) was added, incubated at room temp for 7 min, and the absorption spectrum of the complex determined by a wavelength scan from 330-600 nm using a Ultrospec 2100 Pro spectrophotometer.

### *Cloning and Bacterial Expression of Wild-Type GST-β1 GBD Fusion Protein*

DNA encoding the GBD of the rat β1 subunit (residues 70 –172) was amplified by PCR from a pcDNA3 plasmid encoding rat β1 using primers incorporating *SalI* and *EcoR1* restriction sites (Table S1). The *Sal1/EcoR1* digested product was cloned into the vector pGEX6P2. The resulting plasmid was used to transform BL21 *E. coli* cells. Expression was induced, after growth in LB ampicillin medium at 37°C to an $A_{600}$ of 0.6, using 1 mM IPTG. Cells were incubated at 37°C for a further 4 hr prior to harvesting (6000 rpm; 20 min; 4°C). The cell pellet was lysed by rapid freezing and grinding to a fine powder in liquid $N_2$. The lysate was resuspended in a minimum vol of sucrose buffer (50 mM Tris/HCl, pH 7.5, 0.27 mM sucrose, 1 mM sodium vanadate, 1 mM EDTA, 1 mM EGTA, 10 mM Na β-glycerophosphate, 50 mM NaF, 1 mM DTT, plus one EDTA-free protease inhibitor cocktail tablet per 50 ml). Cell debris was cleared by centrifugation (45000 rpm; 40 min; 4°C). The supernatant was loaded onto a 5ml GSTrap FF column pre-equilibrated with sucrose buffer. The column was washed with 25 ml of sucrose buffer followed by 25 ml of wash buffer (50 mM Tris/HCl, pH 7.5, 200 mM NaCl, 1 mM DTT). The fusion protein was eluted in 20 x 1 ml fractions in elution buffer (50 mM Tris pH 7.5, 200 mM NaCl , 1 mM DTT plus 20 mM reduced glutathione). Fractions containing GST-GBD were identified by protein content and SDS-PAGE. The fusion protein was dialysed overnight into wash buffer.

### *Generation of Mutants*

Mutations were generated in plasmids using the Stratagene QuikChange directed mutagenesis kit, using the mutagenic primers shown in Table S1. Mutated plasmids were used to transform TOP 10 *E. coli* cells. Plasmid DNA from transformants was purified using Qiagen mini-prep kits and mutations confirmed by DNA sequencing. Large-scale purification of the mutant plasmids from 250 ml of TOP 10 *E. coli* cultures was achieved using the Qiagen Plasmid Maxi Hi-speed kit.

### *Concanavalin A-Sepharose Glycogen Binding Assay*

ConA-Sepharose beads were washed in ConA buffer (67 mM Na Hepes, pH 6.8, 0.2 mM $CaCl_2$, 10 mM $MgCl_2$) and incubated (1:1 by vol) with gentle rotation with 50 mg/ml bovine liver glycogen at 4°C for 1 hr. Glycogen-conjugated beads were washed 3x in 2 vols of ConA buffer. For the binding assay, 50 µl of the ConA-Sepharose:glycogen beads were incubated with 30 µg of protein in a final volume of 250 µl ConA buffer, and 10 µl of the mixture was retained for subsequent analysis. The ConA-Sepharose:glycogen beads and protein were incubated together for 1 hr at 4°C with gentle rotation. The beads were recovered by centrifugation (13,000 rpm; 30 sec) and 10 µl of supernatant retained for subsequent analysis. The beads were then washed 2x in 500 µl of ConA buffer, prior to resuspension in 200 µl buffer. Samples (10 µl) of the original mixture, the supernatant and the resuspended pellet were analyzed by SDS-PAGE.

### *Mild Acid Hydrolysis of Glycogen*

Bovine liver glycogen (10 mg/ml) was incubated with 2 vols 1M trifluoroacetic acid at 100°C for 1 hr. Trifluoroacetic acid was removed using a centrifugal vacuum concentrator. The hydrolysate was resuspended in 500 µl of distilled water and subjected to a further cycle of evaporation and resuspension in distilled water.

### *Purification of Glycogen Hydrolysate on β1-GBD Affinity Column*

The GST-β1 GBD column was prepared as described above (as for bacterial expression of wild-type GST-β1 GBD fusion protein, but omitting the final elution step). The acid hydrolysate of bovine glycogen was applied to the resulting GST-β1 GBD column. The column was washed with 50 ml of water, and fragments that interacted with the GST-β1 GBD were eluted in 15 ml of 1M propanoic acid. The propanoic acid eluate was freeze-dried overnight and resuspended in 500 μl of water.

### *High Performance Anion Exchange Chromatography (HPAEC)*

Samples of acid hydrolysed glycogen and β1-GBD affinity-captured fragments in water were analyzed on a BioLC apparatus at 0.25 ml/min using a CarboPac PA1 column (Dionex, Sunnyvale, CA, USA) equilibrated in 150 mM NaOH (eluant A), coupled to a pulsed amperometric detector. Eluant B was 150 mM NaOH plus 500 mM Na acetate. The elution gradient (using mixtures of eluants A and B) was the following: 5% B at time zero rising to 20% B at 50 min and 100% B at 51 min. Peaks from the HPAEC analysis were collected manually, freeze-dried overnight and resuspended in water.

### *Electrospray Ionization Mass Spectrometry*

To 60 μl of the material from HPAEC, 5 μl of 80% (v/v) acetonitrile plus 2% (v/v) formic acid was added. Small aliquots of this solution were transferred to Waters nanotips (Type F; Millipore, Watford, UK) and analyzed by electrospray ionization–mass spectrometry (ES-MS) in negative ion mode on an ABI Q-StarXL spectrometer (Applied Biosystems, Cambridge, UK). Individual ions were subjected to collision induced dissociation (CID) and tandem mass spectrometry (ES-MS/MS). The product ion spectra were used to identify oligosaccharide species and type.

### *Generation of Phosphorylase Limit Dextrin*

A phosphorylase limit dextrin was made from glycogen using a modification of a previous method (Makino and Omichi, 2006). To 0.5 ml of 40 mg/ml bovine liver glycogen, 5 units of glycogen phosphorylase *a* was added in a final volume of 1 ml of 0.1 M Na phosphate buffer. A mock reaction minus phosphorylase was used as a control. The reaction mixtures were transferred to snake skin dialysis tubing (molecular weight cut-off 10,000, Perbio, Cramlington, UK) and dialysed against 0.5 L of 0.1 M Na phosphate buffer, pH 7.0, at room temp for 24 hr. An additional 5 units of phosphorylase was added to the reaction mixture, and dialysis continued against fresh 0.1M sodium phosphate buffer for another 24 hr. The reactions were transferred to 1.5 ml microcentrifuge tubes and incubated at 100$^{o}$C for 5 min to inactivate phosphorylase. After cooling, precipitated material was removed by centrifugation (13,000 rpm; 10 min). The supernatants were then dialysed against distilled water for 2 hr, with three changes, to remove phosphate. The limit dextrin was precipitated by addition of 1 ml of ice-cold 70% ethanol, and collected by centrifugation (13000 rpm; 10 min). The pellet was air-dried prior to resuspension in water and quantification of glucose content as described for glycogen.

### *Other Analytical Procedures*

Protein content was determined by Coomassie Blue binding (Bradford, 1976). SDS-PAGE was run on 4-12% Bis-Tris gels in a MOPS buffer (Invitrogen). Proteins were transferred to a nitrocellulose membranes using the Xcell II blot module (Invitrogen). The membranes were blocked for 1 hr at room temp in Tris-buffered saline (TBS: 50 mM Tris-HCl, pH 7.5, 150 mM NaCl) + 5% fat-free milk, and washed in 4 x 10 mls of TBS. Antibodies (in 10 mls TBS, 1% fat-free milk, 0.2% (v/v) Tween-20) were added and incubated continued for 1 hr at room temp. Membranes were washed 3 x 5 min with TBS plus 0.2% Tween-20. The membranes were then incubated for 1 hr with sheep antibodies conjugated to IR dye 680 (Molecular Probes). The membranes were finally washed for 3 x 5 min with TBS plus 0.2% Tween-20 and 1 x 5 min in TBS. Membranes were scanned in the 680 nm channel of the Li-COR Odyssey IR imager.

## Supplemental References


Bradford, M.M. (1976). A rapid and sensitive method for the quantitation of microgram quantities of protein utilizing the principle of protein-dye binding. Anal Biochem 72, 248-254.

Davies, S.P., Carling, D., and Hardie, D.G. (1989). Tissue distribution of the AMP-activated protein kinase, and lack of activation by cyclic AMP-dependent protein kinase, studied using a specific and sensitive peptide assay. Eur J Biochem 186, 123-128.

Hardie, D.G., Salt, I.P., and Davies, S.P. (2000). Analysis of the role of the AMP-activated protein kinase in the response to cellular stress. Methods Mol Biol 99, 63-75.

Hawley, S.A., Davison, M., Woods, A., Davies, S.P., Beri, R.K., Carling, D., and Hardie, D.G. (1996). Characterization of the AMP-activated protein kinase kinase from rat liver, and identification of threonine-172 as the major site at which it phosphorylates and activates AMP-activated protein kinase. J Biol Chem 271, 27879-27887.

Krisman, C.R. (1962). A method for the colorimetric estimation of glycogen with iodine. Anal Biochem 4, 17-23.

Makino, Y., and Omichi, K. (2006). Purification of glycogen debranching enzyme from porcine brain: evidence for glycogen catabolism in the brain. Biosci Biotechnol Biochem 70, 907-915.

Scott, J.W., Hawley, S.A., Green, K.A., Anis, M., Stewart, G., Scullion, G.A., Norman, D.G., and Hardie, D.G. (2004). CBS domains form energy-sensing modules whose binding of adenosine ligands is disrupted by disease mutations. J Clin Invest 113, 274-284.

Smythe, C., Villar-Palasi, C., and Cohen, P. (1989). Structural and functional studies on rabbit liver glycogenin. Eur J Biochem 183, 205-209.

Takeda, Y., Guan, H.P., and Preiss, J. (1993). Branching of amylose by the branching isoenzymes of maize endosperm. Carbohydr Res 240, 253-263.

Thornton, C., Snowden, M.A., and Carling, D. (1998). Identification of a novel AMP-activated protein kinase b subunit isoform which is highly expressed in skeletal muscle. J Biol Chem 273, 12443-12450.

Woods, A., Salt, I., Scott, J., Hardie, D.G., and Carling, D. (1996). The a1 and a2 isoforms of the AMP-activated protein kinase have similar activities in rat liver but exhibit differences in substrate specificity in vitro. FEBS Lett 397, 347-351.
